# Bis{2-[2-(1*H*-indol-3-yl)ethyl­imino­meth­yl]phenolato-κ^2^ *N*,*O*}nickel(II) *N*,*N*-dimethyl­formamide disolvate

**DOI:** 10.1107/S1600536808012968

**Published:** 2008-05-10

**Authors:** Hapipah M. Ali, Mohamed Ibrahim Mohamed Mustafa, Mohd. Razali Rizal, Seik Weng Ng

**Affiliations:** a Department of Chemistry, University of Malaya, 50603 Kuala Lumpur, Malaysia

## Abstract

The Ni atom in the title compound, [Ni(C_17_H_15_N_2_O)_2_]·2C_3_H_7_NO, lies on a twofold rotation axis. It is *N*,*O*-chelated by the deprotonated Schiff base 2-[2-(1*H*-indol-3-yl)ethyl­imino­meth­yl]phenolate ligand in a square-planar coordination environment. The mol­ecule is linked to a solvent mol­ecule by an indole–dimethyl­formamide N—H⋯O hydrogen bond.

## Related literature

For the structures of Schiff bases derived from the consensation of 2-(indol-3-yl)ethyl­amine and other substituted salicylaldehydes, see: Ali *et al.* (2007*a* ▶,*b* ▶).
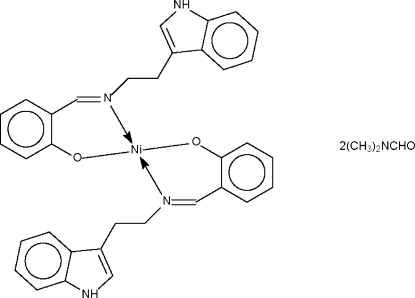


## Experimental

### 

#### Crystal data

[Ni(C_17_H_15_N_2_O)_2_]·2C_3_H_7_NO
*M* *_r_* = 731.52
Monoclinic, 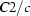

*a* = 38.927 (2) Å
*b* = 5.6999 (3) Å
*c* = 15.7560 (8) Å
β = 98.489 (2)°
*V* = 3457.6 (3) Å^3^
*Z* = 4
Mo *K*α radiation
μ = 0.61 mm^−1^
*T* = 103 (2) K
0.70 × 0.32 × 0.07 mm

#### Data collection

Bruker APEXII diffractometer
Absorption correction: multi-scan (*SADABS*; Sheldrick, 1996 ▶) *T*_min_ = 0.673, *T*_max_ = 0.958
7704 measured reflections
3875 independent reflections
2900 reflections with *I* > 2σ(*I*)
*R*_int_ = 0.030

#### Refinement

*R*[*F* ^2^ > 2σ(*F* ^2^)] = 0.048
*wR*(*F* ^2^) = 0.133
*S* = 1.03
3875 reflections
234 parameters
H-atom parameters constrained
Δρ_max_ = 2.82 e Å^−3^
Δρ_min_ = −0.48 e Å^−3^

### 

Data collection: *APEX2* (Bruker, 2005 ▶); cell refinement: *SAINT* (Bruker, 2005 ▶); data reduction: *SAINT*; program(s) used to solve structure: *SHELXS97* (Sheldrick, 2008 ▶); program(s) used to refine structure: *SHELXL97* (Sheldrick, 2008 ▶); molecular graphics: *X-SEED* (Barbour, 2001 ▶); software used to prepare material for publication: *publCIF* (Westrip, 2008 ▶).

## Supplementary Material

Crystal structure: contains datablocks global, I. DOI: 10.1107/S1600536808012968/sg2223sup1.cif

Structure factors: contains datablocks I. DOI: 10.1107/S1600536808012968/sg2223Isup2.hkl

Additional supplementary materials:  crystallographic information; 3D view; checkCIF report

## Figures and Tables

**Table:** 

Ni1—O1	1.829 (2)
Ni1—N1	1.922 (2)

**Table:** 

O1—Ni1—N1	92.81 (9)
O1—Ni1—N1^i^	87.19 (9)

Symmetry code: (i) 
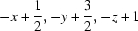
.

**Table 2:** Hydrogen-bond geometry (Å, °)

*D*—H⋯*A*	*D*—H	H⋯*A*	*D*⋯*A*	*D*—H⋯*A*
N3—H3n⋯O2	0.88	1.97	2.811 (3)	159

**Fig. 1.**
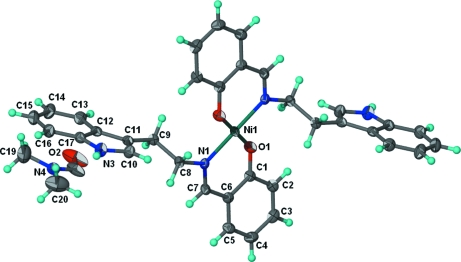
Thermal ellipsoid plot of Ni(C17H14N2O)2.2DMF; displacement ellipsoids are drawn at the 70% probability level, and H atoms are shown as spheres of arbitrary radii. Hydrogen bonds are shown as dashed lines.

## supplementary crystallographic information

### Comment

We have reported a number of metal complexes of Schiff bases derived from the condensation of salicylaldehyde and a biologically active primary amine. The structure of the presence Schiff base, has not been reported, but it is likely to exist as a zwitterion, 2-{[3-(1*H*-indol-3-yl)-propenyl]ammonio}phenolate as 2-{[3-(1*H*-Indol-3-yl)-propenyl]methylammonio}phenolate, synthesized from 2-(indol-3-yl)ethylamine and 2-hydroxy-5-methylacetophenone, exists in this form (Ali *et al.*, 2007*a*, 2007*b*). The nickel derivative crystallizes from DMF as a disolvate (Scheme I, Fig. 1). The metal atom is *N*,*O*-chelated by the deprotonated Schiff base in a square planar coordination enviroment. The molecule is linked to the solvent molecule by an *N*–H_indole_···O_DMF_ hydrogen bond.

### Experimental

Tryptamine (0.30 g, 1.87 mmol) and salicylaldehyde (0.23 g, 1.86 mmol) were heated in ethanol (50 ml) for an hour. The solvent was removed to give the Schiff base. The ligand (0.49 g, 1.91 mmol) and nickel acetate tetrahydrate (0.23 g, 0.93 mmol) were reacted in ethanol (50 ml); several drops of triethylamine were also added. The solvent was removed and the product was recrystallized from DMF.

### Refinement

The carbon-bound H atoms were placed at calculated positions (C—H = 0.95–0.98 Å) and were included in the refinement in the riding-model approximation, with *U*(H) set to 1.2–1.5*U*_eq_(C). The amino H atom also similarly generated [N—H = 0.88Å and *U*(H) = 1.2*U*_eq_(N)].

### Crystal data

**Table:**

[Ni(C_17_H_14_N_2_O)_2_]·2C_3_H_7_NO	*F*_000_ = 1544
*M**_r_* = 731.52	*D*_x_ = 1.405 Mg m^−^^3^
Monoclinic, *C*2/*c*	Mo *K*α radiation λ = 0.71073 Å
Hall symbol: -C 2yc	Cell parameters from 2399 reflections
*a* = 38.927 (2) Å	θ = 2.6–28.5º
*b* = 5.6999 (3) Å	µ = 0.61 mm^−^^1^
*c* = 15.7560 (8) Å	*T* = 103 (2) K
β = 98.489 (2)º	Plate, yellow
*V* = 3457.6 (3) Å^3^	0.70 × 0.32 × 0.07 mm
*Z* = 4	

### Data collection

**Table:**

Bruker APEXII diffractometer	3875 independent reflections
Radiation source: medium-focus sealed tube	2900 reflections with *I* > 2σ(*I*)
Monochromator: graphite	*R*_int_ = 0.030
*T* = 103(2) K	θ_max_ = 27.5º
φ and ω scans	θ_min_ = 2.6º
Absorption correction: Multi-scan(SADABS; Sheldrick, 1996)	*h* = −48→50
*T*_min_ = 0.673, *T*_max_ = 0.958	*k* = −7→5
7704 measured reflections	*l* = −19→20

### Refinement

**Table:**

Refinement on *F*^2^	Secondary atom site location: difference Fourier map
Least-squares matrix: full	Hydrogen site location: inferred from neighbouring sites
*R*[*F*^2^ > 2σ(*F*^2^)] = 0.048	H-atom parameters constrained
*wR*(*F*^2^) = 0.133	*w* = 1/[σ^2^(*F*_o_^2^) + (0.0586*P*)^2^ + 9.176*P*] where *P* = (*F*_o_^2^ + 2*F*_c_^2^)/3
*S* = 1.03	(Δ/σ)_max_ = 0.001
3875 reflections	Δρ_max_ = 0.82 e Å^−^^3^
234 parameters	Δρ_min_ = −0.48 e Å^−^^3^
Primary atom site location: structure-invariant direct methods	Extinction correction: none

### Special details

**Table:**

Experimental. A medium-focus collimator of 0.8 mm diameter was used on the diffractometer to measure the somewhat large crystal.

### Fractional atomic coordinates and isotropic or equivalent isotropic displacement parameters (Å^2^)

**Table:**

	*x*	*y*	*z*	*U*_iso_*/*U*_eq_	
Ni1	0.2500	0.7500	0.5000	0.01379 (15)	
O1	0.27749 (5)	0.8616 (4)	0.42443 (12)	0.0208 (5)	
O2	0.08509 (9)	−0.4015 (5)	0.56375 (17)	0.0564 (9)	
N1	0.23434 (6)	0.4881 (4)	0.42765 (14)	0.0151 (5)	
N3	0.11380 (6)	−0.0268 (5)	0.48184 (16)	0.0224 (6)	
H3N	0.1102	−0.1570	0.5092	0.027*	
N4	0.06308 (7)	−0.7205 (4)	0.62051 (16)	0.0215 (5)	
C1	0.29181 (7)	0.7412 (5)	0.36804 (16)	0.0169 (5)	
C2	0.32195 (8)	0.8312 (5)	0.34033 (17)	0.0199 (6)	
H2	0.3311	0.9776	0.3618	0.024*	
C3	0.33829 (8)	0.7079 (5)	0.28206 (18)	0.0215 (6)	
H3	0.3589	0.7688	0.2650	0.026*	
C4	0.32486 (8)	0.4956 (6)	0.24797 (18)	0.0229 (6)	
H4	0.3360	0.4139	0.2071	0.027*	
C5	0.29539 (8)	0.4055 (5)	0.27382 (17)	0.0202 (6)	
H5	0.2860	0.2618	0.2501	0.024*	
C6	0.27889 (7)	0.5234 (5)	0.33495 (17)	0.0168 (6)	
C7	0.24926 (7)	0.4181 (5)	0.36379 (16)	0.0165 (6)	
H7	0.2396	0.2842	0.3332	0.020*	
C8	0.20526 (7)	0.3382 (5)	0.44577 (18)	0.0169 (6)	
H8A	0.2087	0.2998	0.5077	0.020*	
H8B	0.2058	0.1892	0.4137	0.020*	
C9	0.16973 (7)	0.4520 (5)	0.42173 (17)	0.0171 (6)	
H9A	0.1683	0.5944	0.4570	0.021*	
H9B	0.1666	0.4997	0.3607	0.021*	
C10	0.14497 (8)	0.0886 (5)	0.48646 (19)	0.0211 (6)	
H10	0.1660	0.0388	0.5201	0.025*	
C11	0.14169 (7)	0.2850 (5)	0.43600 (17)	0.0175 (6)	
C12	0.10598 (7)	0.2930 (5)	0.39755 (17)	0.0175 (6)	
C13	0.08600 (8)	0.4471 (6)	0.34154 (18)	0.0229 (7)	
H13	0.0964	0.5807	0.3197	0.028*	
C14	0.05115 (8)	0.4034 (6)	0.31834 (19)	0.0286 (7)	
H14	0.0375	0.5091	0.2808	0.034*	
C15	0.03524 (8)	0.2044 (6)	0.3492 (2)	0.0283 (8)	
H15	0.0111	0.1779	0.3319	0.034*	
C16	0.05416 (8)	0.0488 (6)	0.40391 (19)	0.0249 (7)	
H16	0.0435	−0.0851	0.4248	0.030*	
C17	0.08943 (8)	0.0937 (5)	0.42782 (17)	0.0192 (6)	
C18	0.08801 (10)	−0.5798 (7)	0.6027 (2)	0.0379 (9)	
H18	0.1111	−0.6275	0.6237	0.045*	
C19	0.02741 (10)	−0.6713 (8)	0.5891 (3)	0.0472 (10)	
H19A	0.0258	−0.5257	0.5557	0.071*	
H19B	0.0145	−0.6537	0.6376	0.071*	
H19C	0.0175	−0.8008	0.5525	0.071*	
C20	0.07081 (13)	−0.9334 (7)	0.6694 (3)	0.0521 (11)	
H20A	0.0960	−0.9503	0.6844	0.078*	
H20B	0.0615	−1.0684	0.6350	0.078*	
H20C	0.0602	−0.9258	0.7220	0.078*	

### Atomic displacement parameters (Å^2^)

**Table:**

	*U*^11^	*U*^22^	*U*^33^	*U*^12^	*U*^13^	*U*^23^
Ni1	0.0150 (3)	0.0131 (3)	0.0133 (2)	−0.0029 (2)	0.00219 (17)	−0.0008 (2)
O1	0.0248 (11)	0.0184 (11)	0.0203 (10)	−0.0039 (9)	0.0075 (8)	−0.0011 (9)
O2	0.105 (3)	0.0285 (15)	0.0453 (15)	−0.0269 (16)	0.0436 (16)	−0.0058 (13)
N1	0.0133 (12)	0.0132 (12)	0.0182 (11)	−0.0002 (9)	−0.0001 (9)	0.0026 (9)
N3	0.0214 (13)	0.0202 (13)	0.0256 (13)	−0.0042 (11)	0.0038 (10)	0.0072 (11)
N4	0.0240 (13)	0.0171 (13)	0.0244 (12)	−0.0027 (11)	0.0074 (10)	0.0012 (10)
C1	0.0200 (14)	0.0164 (13)	0.0135 (12)	0.0025 (13)	−0.0003 (10)	0.0036 (12)
C2	0.0231 (15)	0.0189 (14)	0.0174 (13)	−0.0020 (12)	0.0025 (11)	0.0020 (11)
C3	0.0193 (14)	0.0256 (17)	0.0199 (13)	0.0008 (12)	0.0042 (11)	0.0095 (12)
C4	0.0265 (16)	0.0245 (16)	0.0186 (13)	0.0070 (13)	0.0063 (12)	0.0017 (12)
C5	0.0252 (16)	0.0189 (15)	0.0163 (13)	0.0008 (12)	0.0025 (11)	−0.0004 (11)
C6	0.0179 (14)	0.0167 (14)	0.0154 (12)	0.0020 (12)	0.0015 (10)	0.0022 (11)
C7	0.0180 (14)	0.0171 (14)	0.0128 (12)	0.0009 (11)	−0.0027 (10)	0.0005 (11)
C8	0.0168 (14)	0.0147 (13)	0.0191 (13)	−0.0041 (11)	0.0023 (11)	−0.0006 (11)
C9	0.0178 (14)	0.0170 (14)	0.0163 (13)	−0.0013 (12)	0.0019 (10)	0.0017 (11)
C10	0.0183 (15)	0.0207 (15)	0.0242 (14)	−0.0010 (12)	0.0027 (11)	0.0032 (12)
C11	0.0192 (14)	0.0176 (15)	0.0161 (12)	−0.0006 (11)	0.0039 (10)	−0.0022 (11)
C12	0.0204 (14)	0.0193 (15)	0.0136 (12)	−0.0013 (11)	0.0052 (10)	−0.0027 (11)
C13	0.0233 (16)	0.0268 (17)	0.0184 (14)	0.0003 (13)	0.0020 (12)	0.0017 (12)
C14	0.0201 (16)	0.042 (2)	0.0217 (14)	0.0034 (14)	−0.0018 (12)	0.0025 (14)
C15	0.0197 (15)	0.040 (2)	0.0250 (15)	−0.0045 (14)	0.0029 (12)	−0.0057 (14)
C16	0.0205 (16)	0.0300 (18)	0.0255 (15)	−0.0066 (13)	0.0074 (12)	−0.0040 (13)
C17	0.0198 (15)	0.0189 (15)	0.0195 (13)	−0.0034 (12)	0.0050 (11)	−0.0020 (12)
C18	0.043 (2)	0.037 (2)	0.0393 (19)	−0.0171 (17)	0.0231 (16)	−0.0175 (17)
C19	0.029 (2)	0.064 (3)	0.047 (2)	−0.0047 (19)	0.0017 (17)	−0.010 (2)
C20	0.081 (3)	0.032 (2)	0.045 (2)	0.012 (2)	0.016 (2)	0.0071 (19)

### Geometric parameters (Å, °)

**Table:**

Ni1—O1^i^	1.829 (2)	C8—C9	1.524 (4)
Ni1—O1	1.829 (2)	C8—H8A	0.9900
Ni1—N1^i^	1.922 (2)	C8—H8B	0.9900
Ni1—N1	1.922 (2)	C9—C11	1.490 (4)
O1—C1	1.310 (3)	C9—H9A	0.9900
O2—C18	1.184 (5)	C9—H9B	0.9900
N1—C7	1.297 (3)	C10—C11	1.368 (4)
N1—C8	1.479 (3)	C10—H10	0.9500
N3—C17	1.363 (4)	C11—C12	1.433 (4)
N3—C10	1.373 (4)	C12—C13	1.397 (4)
N3—H3N	0.8800	C12—C17	1.423 (4)
N4—C18	1.320 (4)	C13—C14	1.375 (4)
N4—C19	1.431 (4)	C13—H13	0.9500
N4—C20	1.445 (4)	C14—C15	1.412 (5)
C1—C2	1.408 (4)	C14—H14	0.9500
C1—C6	1.410 (4)	C15—C16	1.373 (5)
C2—C3	1.383 (4)	C15—H15	0.9500
C2—H2	0.9500	C16—C17	1.393 (4)
C3—C4	1.394 (4)	C16—H16	0.9500
C3—H3	0.9500	C18—H18	0.9500
C4—C5	1.372 (4)	C19—H19A	0.9800
C4—H4	0.9500	C19—H19B	0.9800
C5—C6	1.405 (4)	C19—H19C	0.9800
C5—H5	0.9500	C20—H20A	0.9800
C6—C7	1.432 (4)	C20—H20B	0.9800
C7—H7	0.9500	C20—H20C	0.9800
			
O1^i^—Ni1—O1	180.00 (10)	C11—C9—H9A	109.6
O1^i^—Ni1—N1^i^	92.81 (9)	C8—C9—H9A	109.6
O1—Ni1—N1^i^	87.19 (9)	C11—C9—H9B	109.6
O1^i^—Ni1—N1	87.19 (9)	C8—C9—H9B	109.6
O1—Ni1—N1	92.81 (9)	H9A—C9—H9B	108.1
N1^i^—Ni1—N1	180.000 (1)	C11—C10—N3	110.8 (3)
C1—O1—Ni1	127.41 (19)	C11—C10—H10	124.6
C7—N1—C8	114.6 (2)	N3—C10—H10	124.6
C7—N1—Ni1	124.0 (2)	C10—C11—C12	105.9 (2)
C8—N1—Ni1	121.26 (17)	C10—C11—C9	127.1 (3)
C17—N3—C10	108.6 (2)	C12—C11—C9	127.0 (2)
C17—N3—H3N	125.7	C13—C12—C17	118.4 (3)
C10—N3—H3N	125.7	C13—C12—C11	134.6 (3)
C18—N4—C19	120.9 (3)	C17—C12—C11	107.0 (2)
C18—N4—C20	121.4 (3)	C14—C13—C12	119.3 (3)
C19—N4—C20	117.6 (3)	C14—C13—H13	120.3
O1—C1—C2	118.5 (3)	C12—C13—H13	120.3
O1—C1—C6	123.2 (2)	C13—C14—C15	121.3 (3)
C2—C1—C6	118.3 (3)	C13—C14—H14	119.3
C3—C2—C1	120.4 (3)	C15—C14—H14	119.3
C3—C2—H2	119.8	C16—C15—C14	120.9 (3)
C1—C2—H2	119.8	C16—C15—H15	119.6
C2—C3—C4	120.9 (3)	C14—C15—H15	119.6
C2—C3—H3	119.5	C15—C16—C17	117.9 (3)
C4—C3—H3	119.5	C15—C16—H16	121.1
C5—C4—C3	119.5 (3)	C17—C16—H16	121.1
C5—C4—H4	120.2	N3—C17—C16	130.1 (3)
C3—C4—H4	120.2	N3—C17—C12	107.7 (3)
C4—C5—C6	120.7 (3)	C16—C17—C12	122.2 (3)
C4—C5—H5	119.6	O2—C18—N4	127.9 (4)
C6—C5—H5	119.6	O2—C18—H18	116.1
C5—C6—C1	120.0 (3)	N4—C18—H18	116.1
C5—C6—C7	119.2 (3)	N4—C19—H19A	109.5
C1—C6—C7	120.8 (2)	N4—C19—H19B	109.5
N1—C7—C6	126.2 (3)	H19A—C19—H19B	109.5
N1—C7—H7	116.9	N4—C19—H19C	109.5
C6—C7—H7	116.9	H19A—C19—H19C	109.5
N1—C8—C9	113.5 (2)	H19B—C19—H19C	109.5
N1—C8—H8A	108.9	N4—C20—H20A	109.5
C9—C8—H8A	108.9	N4—C20—H20B	109.5
N1—C8—H8B	108.9	H20A—C20—H20B	109.5
C9—C8—H8B	108.9	N4—C20—H20C	109.5
H8A—C8—H8B	107.7	H20A—C20—H20C	109.5
C11—C9—C8	110.4 (2)	H20B—C20—H20C	109.5
			
N1^i^—Ni1—O1—C1	153.4 (2)	N1—C8—C9—C11	176.0 (2)
N1—Ni1—O1—C1	−26.6 (2)	C17—N3—C10—C11	0.0 (3)
O1^i^—Ni1—N1—C7	−164.6 (2)	N3—C10—C11—C12	0.2 (3)
O1—Ni1—N1—C7	15.4 (2)	N3—C10—C11—C9	−178.0 (3)
O1^i^—Ni1—N1—C8	10.6 (2)	C8—C9—C11—C10	19.2 (4)
O1—Ni1—N1—C8	−169.4 (2)	C8—C9—C11—C12	−158.7 (3)
Ni1—O1—C1—C2	−155.2 (2)	C10—C11—C12—C13	179.0 (3)
Ni1—O1—C1—C6	23.5 (4)	C9—C11—C12—C13	−2.8 (5)
O1—C1—C2—C3	178.8 (3)	C10—C11—C12—C17	−0.3 (3)
C6—C1—C2—C3	0.0 (4)	C9—C11—C12—C17	177.9 (3)
C1—C2—C3—C4	1.6 (4)	C17—C12—C13—C14	0.8 (4)
C2—C3—C4—C5	−1.3 (4)	C11—C12—C13—C14	−178.5 (3)
C3—C4—C5—C6	−0.8 (4)	C12—C13—C14—C15	−0.7 (5)
C4—C5—C6—C1	2.4 (4)	C13—C14—C15—C16	0.4 (5)
C4—C5—C6—C7	−176.7 (3)	C14—C15—C16—C17	0.0 (4)
O1—C1—C6—C5	179.3 (3)	C10—N3—C17—C16	−178.7 (3)
C2—C1—C6—C5	−2.0 (4)	C10—N3—C17—C12	−0.2 (3)
O1—C1—C6—C7	−1.6 (4)	C15—C16—C17—N3	178.4 (3)
C2—C1—C6—C7	177.1 (3)	C15—C16—C17—C12	0.1 (4)
C8—N1—C7—C6	−177.0 (3)	C13—C12—C17—N3	−179.1 (2)
Ni1—N1—C7—C6	−1.5 (4)	C11—C12—C17—N3	0.3 (3)
C5—C6—C7—N1	169.8 (3)	C13—C12—C17—C16	−0.4 (4)
C1—C6—C7—N1	−9.3 (4)	C11—C12—C17—C16	179.0 (3)
C7—N1—C8—C9	−108.6 (3)	C19—N4—C18—O2	2.6 (5)
Ni1—N1—C8—C9	75.7 (3)	C20—N4—C18—O2	−179.7 (3)

Symmetry codes: (i) −*x*+1/2, −*y*+3/2, −*z*+1.

### Hydrogen-bond geometry (Å, °)

**Table:**

*D*—H···*A*	*D*—H	H···*A*	*D*···*A*	*D*—H···*A*
N3—H3N···O2	0.88	1.97	2.811 (3)	159
